# Hepatic pseudocystic metastasis of well-differentiated ileal neuroendocrine tumor: a case report with review of the literature

**DOI:** 10.1186/1746-1596-8-148

**Published:** 2013-09-13

**Authors:** Stefano Fiori, Alessandro Del Gobbo, Gabriella Gaudioso, Lucio Caccamo, Sara Massironi, Federica Cavalcoli, Silvano Bosari, Stefano Ferrero

**Affiliations:** 1 Division of Pathology, Fondazione IRCCS “Ca’ Granda” - Ospedale Maggiore Policlinico, Via Francesco Sforza 35, Milano 20100, Italy; 2 Liver Transplant Unit, Fondazione IRCCS “Ca’ Granda” Ospedale Maggiore Policlinico, Milan, Italy; 3 Gastroenterology Unit II, Fondazione IRCCS “Ca’ Granda” Ospedale Maggiore Policlinico, Milan, Italy; 4 Department of Clinical/Surgical Pathophysiology and Organ Transplant, University of Milan Medical School, Milan, Italy; 5 Department of Biomedical, Surgical and Dental Sciences, University of Milan Medical School, Milan, Italy

**Keywords:** Pseudocystic metastasis, Neuroendocrine tumor, Hepatic malignancies

## Abstract

**Abstract:**

Imaging appearance of cyst-like changes is most frequently described in primary neuroendocrine lesions, especially pancreatic NETs.

The imaging finding of a pseudocystic lesion of the liver puts in differential diagnosis many pathologies such as infectious diseases, simple biliary cysts up to biliary cystadenomas and eventually to primary or metastatic malignancies.

Primary or metastatic hepatic malignancies with pseudocystic aspects are rare, and a pseudocystic aspect is reported only after neo-adjuvant treatment.

Liver metastasis of untreated neuroendocrine tumors are usually solid and, to our knowledge, only two cases of neuroendocrine cystic hepatic metastases of ileal atypical carcinoids have been reported so far.

We present a case of a 67 years old man with synchronous finding of an untreated hepatic pseudocystic lesion and an ileal mass histologically diagnosed as a well differentiated (G1) neuroendocrine tumor.

**Virtual slides:**

The virtual slides for this article can be found here: http://www.diagnosticpathology.diagnomx.eu/vs/1443883503102967.

## Introduction

Gastro-entero-pancreatic neuroendocrine tumors (NETs) are uncommon, accounting for around 2% of all gastrointestinal tumors [1-3], and are characterized by disparate clinical and pathological features. Their incidence has been on the rise over the last two decades; this, along with an overall good prognostic expectancy, explains the relatively high prevalence estimate of 35/100.000 [4].

This family of heterogeneous neoplasms is thought to derive from the gastrointestinal diffuse endocrine system, and includes functioning tumors, which secrete a variety of peptide hormones with the resulting clinical syndromes, and non-functioning tumors. The lattest are often metastatic at the time of diagnosis. Even if the majority of NETs are well-differentiated, low-grade tumors, others may show an aggressive, frankly malignant behavior. Histopathological grading of these lesions has been recently reviewed: the 2010 WHO classification acknowledges and emphasizes the malignant potential of neuroendocrine neoplasms [5]. According to this classification, based on the tumor’s proliferative features (mitotic count and Ki67 proliferation index) three grades are identified, and they are illustrated in Table 1.

**Table 1:** **Histopathological characteristics of neuroendocrine carcinomas of the small intestine**[5]

**Histotype**	**Mitoses**	**Ki67 index**	**Nuclear atypia**	**Necrosis**	**Immunoreactivity**
Well differentiated (G1- carcinoids)	< 2/10 HPF	< 2%	Absent	Absent	Synaptophysin (+)
					Chromogranin A (+)
Moderately differentiated (G2)	2–20/10 HPF	3-20%	May be present	May be present	Synaptophysin (+)
					Chromogranin A (+)
Poorly differentiated (G3 - neuroendocrine carcinoma)	> 20/10 HPF	> 20%	Usually present	Usually present	Synaptophysin (+)
					Chromogranin A (−/+)

Ki67 labeling index cut off of 3% allows the division of NETs in well-differentiated and moderately differentiated and it predicts metatasis or recurrence [6].

Synaptophysin and Chromogranin A are the most useful markers to differentiate NETs from non-endocrine poorly differentiated adenocarcinoma [7].

Small bowel is the most common site of presentation of gastrointestinal NETs (44.7%), followed by rectum (19.6%), colon (17%), appendix (16.7%), pancreas (12.1%) and stomach (8.9%) [8].

The most frequent site of metastases of gastrointestinal NETs, apart from regional lymphnodes, is the liver: hepatic metastases are found at the time of diagnosis in up to 40% of ileal and 80% of caecal lesions [9]. Furthermore, 59–80% of patients with pancreatic non-insulinoma tumors bear synchronous liver metastases [10]. In a minority (5–14%) of patients with NET liver metastases, the primary tumor can’t be identified.

An aggressive surgical management of neuroendocrine hepatic metastases has been demonstrated to improve 5-years survival rates [11], hence the importance of an accurate histological diagnosis.

Liver metastases are usually solid with a dense capillary network; thereby, computerized tomography (CT) and magnetic resonance (MR) scans reveal hypervascularization with arterial phase enhancement.

A small minority of hepatic NET metastases have a cystic appearance at standard cross-sectional scans, and may be mistaken for benign lesions. Cystic changes, due to central tumor necrosis, are described in NET hepatic metastases as a result of chemotherapic treatment.

A primary cystic appearance is exceedingly rare in untreated cases. At the best of our knowledge, only two other cases of cystic hepatic metastases of untreated ileal NETs have been reported [12,13].

We present a case of a 67 years old man with synchronous finding of a hepatic pseudocystic lesion and an ileal mass, histologically diagnosed as a well differentiated (G1) NET.

## Case presentation

### Clinical presentation

A 67 years old man was referred to our attention for an incidental finding (during routine abdominal ultrasonography) of four hepatic lesions. He had a history of hypertension, ischemic cardiomyopathy, chronic obstructive pulmonary disease and prostatic nodular hyperplasia.

### Diagnostics

Ultrasonography showed that three lesions, in segments III and VII, were solid, less than 2 cm in diameter; the fourth one, in segment VII, was a 9 cm multilocular cyst with mixed echostructure (hyperechogenic with fluid content).

**Figure 1:**
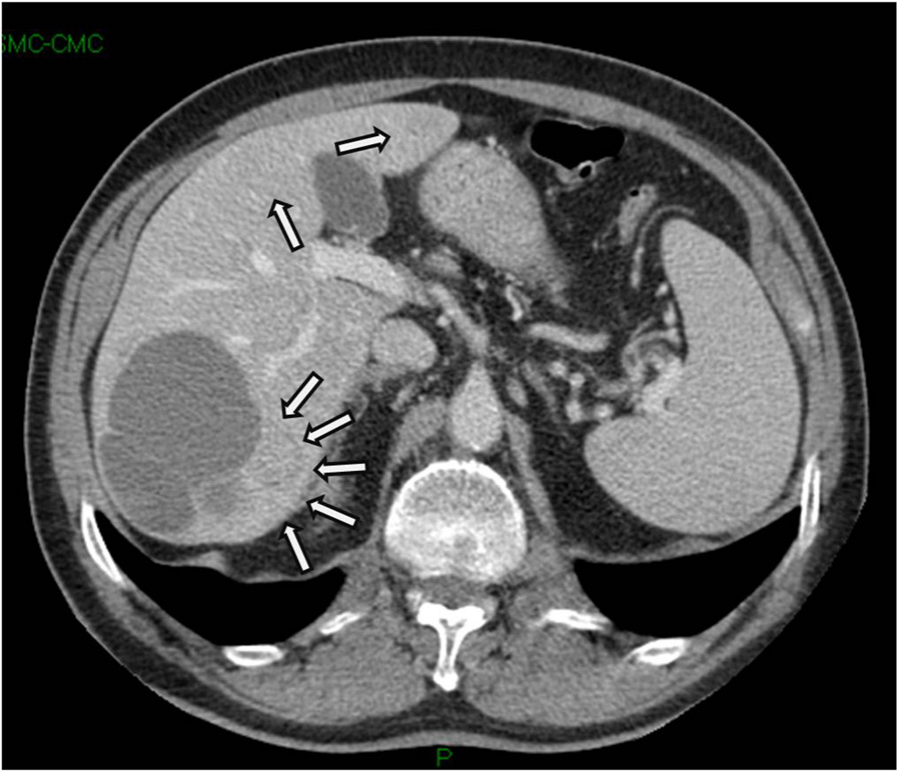
Computed tomography portal contrast phase image showing the presence of three slightly hypodense nodular lesions (arrows), the larger sited in the medial wall of a large cyst in segment VII.

An abdominal contrast-enhanced CT scan (Figure 1) confirmed all the four lesions, and revealed three further subcentimetric nodules, in hepatic segments II, III and VII.

The cyst showed a portal-phase enhancement in walls and inner septa. Moreover, a 3 cm thickening of terminal ileum wall, and an enlarged (cm 1.5) mesenteric lymphnode were highlighted.

A pan-colonoscopy confirmed the ileo-caecal mass. Histological examination of the endoscopic biopsy specimens was consistent with a well differentiated (G1) NET. Immunostaining for both Synaptophysin and Chromogranin A turned out positive. Afterwards, a fine-needle biopsy of a III segment nodular lesion was performed: histological diagnosis was of well-differentiated (G1) NET hepatic metastasis.

**Figure 2:**
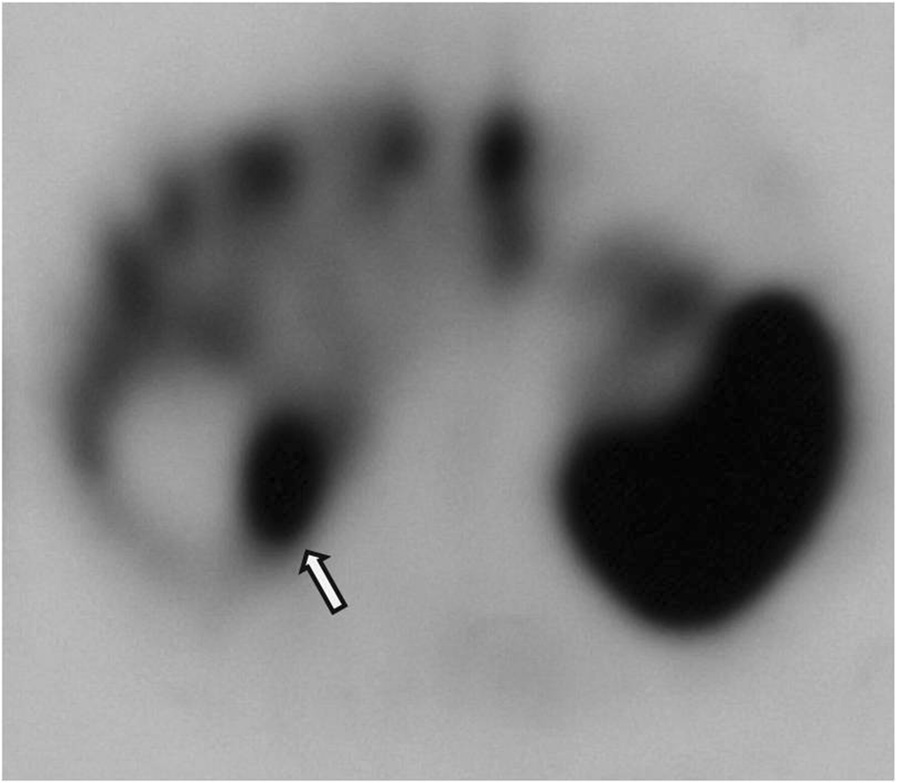
^**111**^**Indium-pentetreotide scintigraphy showing multiple intrahepatic hypercaptations; the more posterior of them (arrow) is near to a large cyst sited in segment VII.**

Plasma Chromogranin A levels were evaluated, and turned out raised (1096 U/I; r.v. <33), as well as 5-indolacetic acid urinary excretion (58.9 mg/g creatinine; r.v. 2.0-10.0). Clinical staging was concluded with a ^111^In-pentetreotide somatostatin receptor scintigraphy, which displayed intestinal and plurifocal hepatic hypercaptation (Figure 2).

### Surgery

The patient underwent a synchronous right hemicolectomy, left hepatic lobectomy, VII segment resection and cholecystectomy with concurrent radiofrequency ablation of the subcentimetric hepatic nodules. Postoperative course was regular, and a medical regimen of somatostatin analogs (SSAs) was started.

### Gross examination and histology

**Figure 3:**
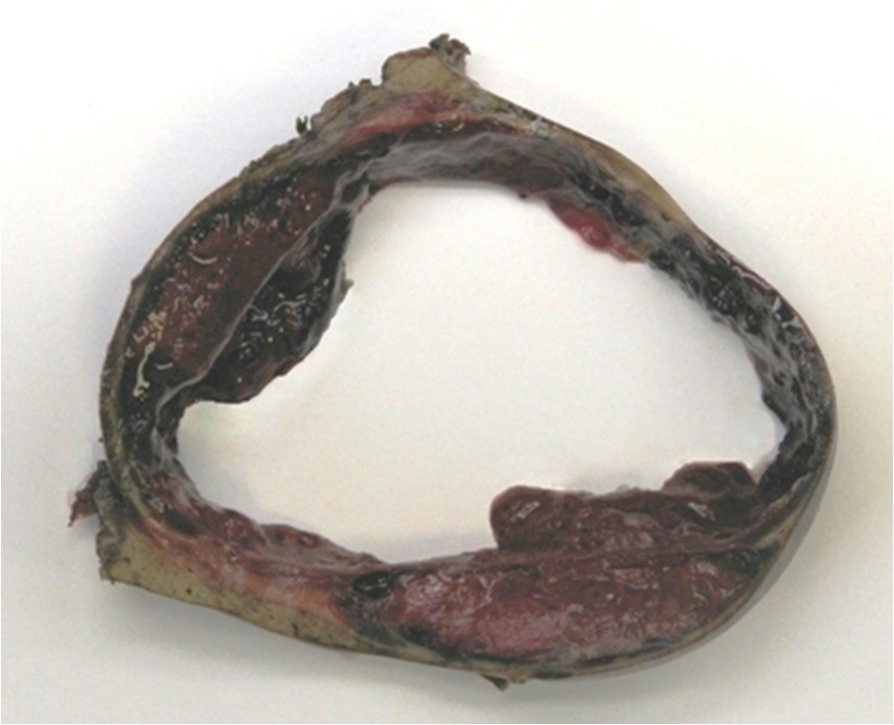
Gross view of the hepatic pseudocystic metastasis specimen, showing the peripheral solid wall, the bloody content and with the surrounding normal hepatic parenchyma.

Gross examination of the resected specimens revealed a 2.5 cm peduncolated mass in the terminal ileum, a 14 cm thick-wall pseudocystic lesion with bloody content in VII hepatic segment (Figure 3), and several small (1.4 cm the largest), whitish nodules in the left hepatic lobe. The gallbladder contained yellowish gallstones.

**Figure 4:**
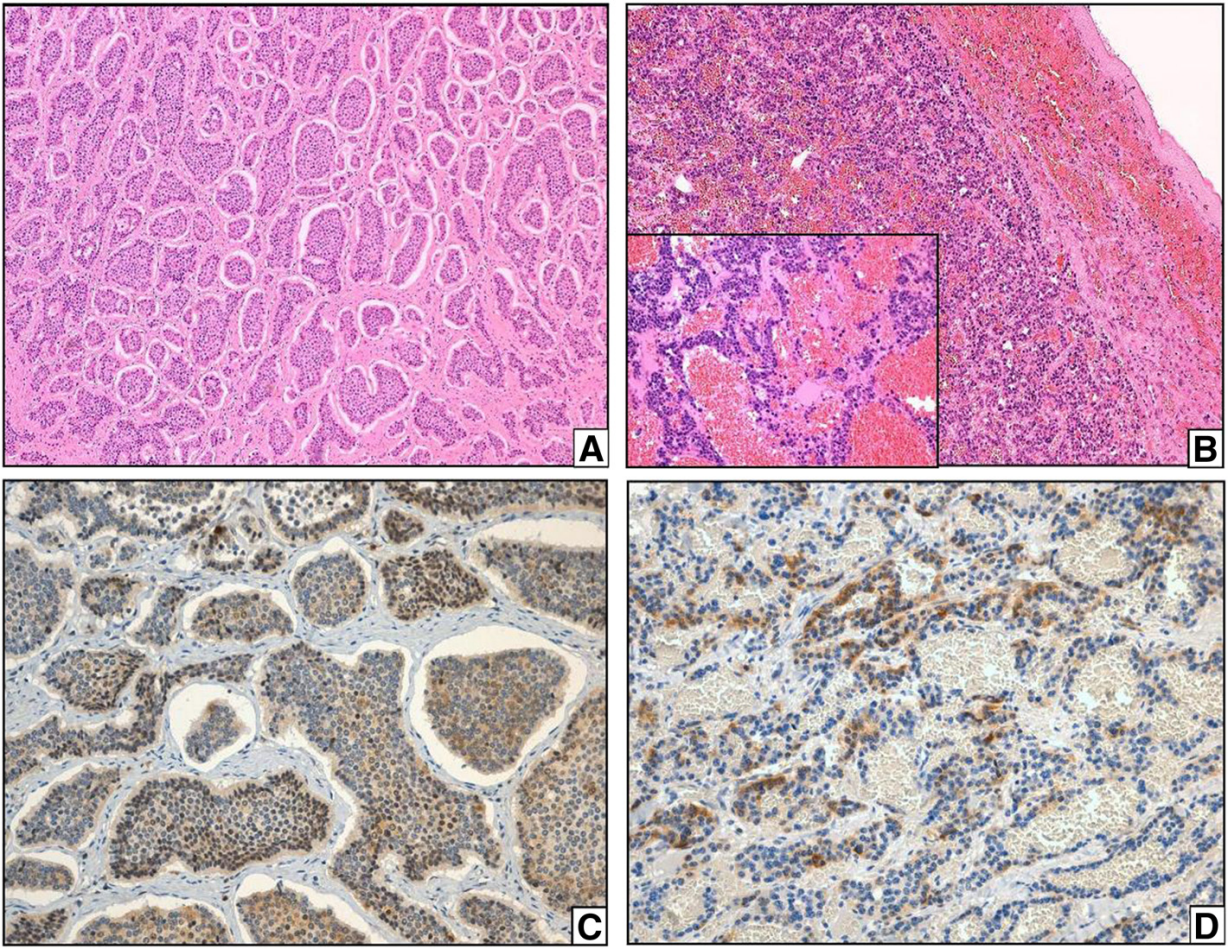
**Blood-filled core and delicate septa. (A)** Microscopic view of the primary ileal tumor, composed of rounded nests of closely packed, monomorphous cells (EE, 10×). **(B)** The same architectural pattern of the hepatic metastasis, with the central, blood-filled core of blood and delicate septa of tumoral cells (EE; 10x). **(C-D)** Granular, cytoplasmic immunostaining for serotonin in both primary and metastatic tumors (20×).

Microscopically, the ileal tumor was composed of rounded nests of closely packed, monomorphous cells lacking atypia, and with a peripheral-palisading pattern of growth. The tumor exhibited infiltration of perivisceral adipose tissue, along with two mesenteric nodal metastases (Figure 4A). The pseudocystic hepatic lesion showed two different components: a peripheral thick wall, which resembled the ileal tumor, and a blood-filled core, with thin fibrous septa and floating neoplastic cells (Figure 4B). All the other hepatic nodules were consistent with NET metastases. The gallbladder and all the surgical margins were free of disease.

Mitotic count was lower than 2/10 HPF, and Ki67 proliferative index (Ventana, clone 30–9) turned out 1% in both primary and metastatic lesions. All the described lesions showed a positive reaction to Synaptophysin (Ventana, clone MRQ-40) and Chromogranin A (Ventana, clone LK2H10).

Moreover, immunohistochemical stainings for Insulin (DAKO, clone IN-05), Glucagon (DAKO, clone A56501), VIP (Sigma Aldrich, polyclonal) and Serotonin (DAKO, clone 5HT-H209) were performed: only Serotonin stained weakly positive in the ileal mass and in the cystic lesion (Figure 4C and 4D).

According to the 2010 World Health Organization (WHO) Classification of Tumors of the Digestive System [5], the final diagnosis was of well differentiated (G1) neuroendocrine tumor of the ileum, with hepatic and nodal metastases. Pathological stage, in agreement with the TNM classification of malignant tumors (VII edition, 2009) was pT3, N1, M1. Clinical stage was IV.

### Post operative outcome and follow-up

The patient was discharged without complications after fifteen days of recovery. Nine months later he is in good health, with no evidence of residual disease and under a medical regimen of long-acting SSAs.

## Discussion

Hepatic neoplasms with cystic features are rare [14].

The imaging finding of a pseudocystic lesion of the liver, in absence of clinical symptoms, implies several differential diagnoses, ranging from infectious diseases such as echinococcal cysts [15], benign lesions as simple biliary cysts and biliary cystadenomas, up to primary or metastatic malignancies.

Two cases of pseudocystic GEP/NETs liver metastases [12,13] and two primary hepatic pseudocystic NETs [16,17] have been described.

However, this is the first case of a G1 NET that provoked pseudocystic hepatic metastasis. Hepatic pseudocystic secondarisms are also reported from uterine cervical carcinoma [1], spinal haemangiopericytoma [2] and nasopharyngeal carcinoma [3].

Neuroendocrine tumors can undergo pseudocystic metastases in different sites: a cerebral cystic metastasis of neuroendocrine carcinoma, with unknown primary tumor, has been reported [18].

Cystic changes are often reported in liver malignancies after a neo-adjuvant treatment. This is thought to be due to therapy-induced tumor necrosis [14].

A pseudocystic appearance with a peculiar peripheral contrast enhancement are described in pancreatic NETs [15] and in a case of a case of primary micro-neuroendocrine tumor arising in a horseshoe kidney [19].

Primary cystic NETs display a less frequent tendency to undergo nodal and hepatic spread when compared to solid counterparts.

We observed an aggressive behaviour, with multiple hepatic and nodal metastases in a well differentiated (G1) ileal NET at the time of diagnosis.

In our case, once the neuroendocrine nature of the primary ileal mass was histologically confirmed, the ^111^In-pentetreotide somatostatin receptor scintigraphy revealed the same capitation pattern in the hepatic cyst, suggesting its secondary nature. Taking account on these acknowledgements, a complete resection of the cyst was performed. Neo-adjuvant chemotherapy was not performed, despite the advanced clinical stage [20].

Both macroscopic and microscopic examination showed that the pseudocystic appearance of this metastasis was not due to the central necrosis, but to intratumoral haemorrhage.

All the described findings were incidental, and patient did not complain for pain, flushing, diarrhea or other symptoms related to a ‘’hypersecretion syndrome”. For this reason, the hepatic pseudocyst could have been misdiagnosed, unless an ileal mass was identificated and raised the suspicion for a metastatic NET.

A complete resection of NET hepatic metastases leads to an improved prognostic outcome: 5-years overall survival rates higher than 70% [21] are reported, despite recurrences develop in up to 80% of patients [22]. Thus, the rationale to remove NET liver metastases, once technically feasible, is established. In order to either obtain an accurate pre-treatment assessment and plan an adequate surgical strategy, it is crucial to differentiate liver NET metastases from other primary or secondary hepatic malignancies (that may not take advantage of the similar prognostic improvements), as well as from benign lesions. For these reasons, we recommend to keep in consideration unusual NET presentations, such as the described one.

## Consent

Written informed consent was obtained from the patient for publication of this Case Report and any accompanying images. A copy of the written consent is available for review by the Editor-in-Chief of this journal.

## Competing interests

The authors declare that they have no competing interests.

## Authors’ contributions

All authors read approved the manuscript. SFiori, ADG and FC analysed, interpreted the patient’s data and drafted the manuscript. GG performed immunohistochemistry. LC and SR revised the clinical data and supervised the case report. SF and SB revised the pathology data and supervised the case report. All authors read and approved the final manuscript.
